# Higher Neutrophil-to-Lymphocyte Ratio (NLR) Is a Preoperative Inflammation Biomarker of Poor Prognosis in HIV-Infected Patients with Colorectal Cancer: A Retrospective Study

**DOI:** 10.1155/2023/7966625

**Published:** 2023-03-06

**Authors:** Li Deng, Yanhui Si, Qian Wu, Ye Cao, Shixian Lian, Lei Li

**Affiliations:** Department of General Surgery, Shanghai Public Health Clinical Center, Fudan University, Shanghai, China

## Abstract

**Background:**

The serum systemic inflammation biomarkers are known predictors of colorectal cancer (CRC) patient prognosis. However, their significance in human immunodeficiency virus (HIV)-infected patients with CRC has not been studied. To address this gap, we conducted a retrospective study to evaluate the prognostic value of preoperative systemic inflammation biomarkers in HIV-infected patients with CRC.

**Methods:**

The study enrolled 57 patients with colorectal cancer (CRC) and HIV who underwent surgery at the Shanghai Public Health Clinical Center between January 2015 and December 2021. Preoperative tests were conducted, and systemic inflammation biomarkers were measured. The patients were categorized into two groups using the optimal cut-off value. The Kaplan–Meier method and the log-rank test were used to determine overall survival (OS) and progression-free survival (PFS). Multivariate analysis was performed using the Cox proportional regression model. A time-dependent receiver operating characteristic (t-ROC) was used to compare the prognostic abilities of the biomarkers.

**Results:**

The study included 57 HIV-infected CRC patients, with a median age of 60 and a follow-up time ranging from 3 to 86 months. Of the patients, 49 were male and 8 were female. The cumulative three-year OS and PFS rates were 55.0% and 45.0%, respectively. The optimal cut-off value for preoperative NLR was found to be 2.8, which was significantly correlated with lower CD8+ T and CD3+ T lymphocyte counts. Multivariate Cox regression analysis revealed that a low NLR was an independent predictor of better OS and PFS (OS: HR = 0.094, 95% CI: 0.02–0.45, *P*=0.003; PFS: HR = 0.265, 95% CI: 0.088–0.8, *P*=0.019). The time-dependent receiver operating characteristic (t-ROC) analysis showed that NLR was a superior systemic inflammation biomarker for predicting the prognosis of HIV-infected CRC patients throughout the observation period.

**Conclusion:**

The preoperative neutrophil-to-lymphocyte ratio (NLR), an easily measurable immune biomarker, may provide useful prognostic information in HIV-infected colorectal cancer (CRC) patients.

## 1. Introduction

Colorectal cancer (CRC) is a prevalent form of cancer and the leading cause of cancer-related deaths worldwide [1]. The use of combination antiretroviral therapy (cART) has resulted in an increase in CRC incidence in patients infected with the human immunodeficiency virus (HIV) [2]. Furthermore, HIV-positive patients with CRC have a poorer prognosis than HIV-negative patients [2, 3]. While known traditional risk factors for CRC include obesity, excessive red meat consumption, family history, and hereditary polyposis, HIV-induced immunodeficiency is also believed to increase CRC risk [4]. HIV activates CD4^+^ T lymphocytes, dendritic cells, and macrophages, resulting in immune system dysfunction [5]. In HIV-infected CRC patients, a reduction in immune surveillance, increased expression of the immune checkpoint, direct viral protein effects, or cytokine dysregulation may lead to poorer outcomes [6]. Few studies to date have explored the relationship between HIV infection and colorectal cancer (CRC). Moreover, in China, HIV-infected CRC patients often experience poorer outcomes due to insufficient understanding and discriminatory attitudes towards HIV.

Although molecular subtypes had the potential to identify CRC patients with a worse prognosis, the results based on somatic copy number, CpG island hypermethylation, and gene expression made them difficult to use in clinical practice [7, 8]. Another important predictor of prognosis in CRC patients is the cancer-associated systemic inflammation status [9]. The tumor microenvironment includes monocytes, neutrophils, platelets, and lymphocytes. Therefore, serum systemic inflammation biomarkers, such as the neutrophil-to-lymphocyte ratio (NLR), lymphocyte-to-monocyte ratio (LMR), platelet-to-lymphocyte ratio (PLR), systemic inflammation score (SIS), and prognostic nutritional index (PNI), are useful in numerous studies [9]. New biomarkers, including the fibrinogen to prealbumin ratio and the albumin to fibrinogen ratio, have emerged as potential predictors of CRC outcome [10]. HIV infection functionally impairs the HIV-specific CD8^+^ T and CD4^+^ T lymphocytes, resulting in the inability of the host immune system to control HIV [11]. Although cARTs do not completely eradicate HIV, continued treatment can restore the immune system function [12]. A previous study also found no difference in tumor-infiltrating lymphocytes between HIV-positive and HIV-negative CRC patients [13]. Assessing systemic inflammation biomarkers in CRC may be aided by studying immune reconstitution in HIV-infected patients. Early identification of potential predictors in these patients could improve their chances of survival. However, to our knowledge, no research has been conducted to investigate the relationship between serum systemic inflammation biomarkers and HIV-related CRC prognosis. Therefore, the objective of this study is to evaluate the prognostic value of systemic inflammation biomarkers in patients with HIV-related CRC.

## 2. Methods

### 2.1. Patients

This study retrospectively collected data from all human immunodeficiency virus (HIV)-infected patients with colorectal cancer (CRC) who underwent surgery between January 2015 and December 2021 at the Shanghai Public Health Clinical Center (SHPHC). A total of 57 HIV-infected patients were recruited. The inclusion criteria were as follows: preoperatively diagnosed as primary colorectal adenocarcinoma and anal squamous cell carcinoma. The exclusion criteria were as follows: evidence of a severe inflammatory condition; chronic inflammation diseases except HIV infection; incomplete clinicopathological data. The following characteristics were included: gender, age, body mass index (BMI), disease history (hypertension, diabetes, tuberculosis infection, smoking, and alcohol abuse), presence of intestinal obstruction on admission (diagnosis was based on the symptoms of no bowel movements combined with radiographic or colonoscopic findings), duration of HIV infection, and treatment (antiviral drugs), preoperative tests (routine blood tests, liver function, tumor markers, and plasma lipids), tumor location (if the primary tumor was located from cecum to transverse colon, it was defined as right colon cancer, and from splenic flexure of colon to upper rectum, it was defined as left colon cancer, and from middle or lower rectum to anal, it was defined as rectal cancer), postoperative pathological stage (using the eighth AJCC edition), mismatch repair (MMR) status (using immunohistochemical results of four MMR proteins) and surgical resection with/without tumor residuals. This study was conducted according to the Declaration of Helsinki, and the Shanghai Public Health Clinical Center approved it. The patients' data were obtained from the hospital database and used for research purposes.

### 2.2. Follow-Up

Regular follow-up assessments were conducted every three months during the first two years postsurgery and every six months during the following two years. The patients were admitted to the inpatient unit for routine blood tests and enhanced chest and abdominal CT scans. After three years, follow-up assessments were performed annually. Local recurrence, enlargement of unresected tumor lesions, and distant organ metastases were all considered indicators of progression. In the event of patient death, confirmation was obtained either from relevant hospital records or from notification provided by the patient's family during a telephone follow-up. The latest censoring date for survival time evaluation was March 2022. Overall survival (OS) was defined as the time from surgery to death from any cause. Progression-free survival (PFS) was defined as the interval between surgery and either progression or death.

### 2.3. Definition of Inflammation-Related Biomarkers

The laboratory tests from each patient were obtained within 1-week before surgical resection of the primary tumor. The interval between the end of last neoadjuvant treatment and surgery is at least over six weeks. According to a recent review [9], the neutrophil-to-lymphocyte ratio (NLR) was calculated by dividing the absolute neutrophil count by the absolute lymphocyte count. The lymphocyte-to-monocyte ratio (LMR) was defined as the absolute lymphocyte count divided by the monocyte count. The platelet-to-lymphocyte ratio (PLR) was the platelet count divided by the lymphocyte count. The prognostic nutritional index (PNI) was defined as the albumin level (g/L) + 5 × lymphocyte count per liter [14]. The systemic inflammation score (SIS) definition was based on the combination of the preoperative albumin level and lymphocyte-monocyte ratio (LMR) [15], and modified SIS (mSIS) was an improved scoring based on the LMR optimal cut-off value [16]. The detailed scoring of SIS and mSIS is shown in Table S1.

### 2.4. Statistics

Categorical variables were presented as proportions and integers, while continuous variables were reported as medians, means (standard deviations), and maximum ranges. The Wilcoxon rank-sum test was used for continuous variables, and Fisher's exact test was used for categorical variables. To analyze the survival differences, patients were categorized into two groups based on the continuous variables best cut-off value using the “MaxStat” R package (maximally selected rank statistics) [17]. Kaplan–Meier survival curves were constructed according to the group differences, which were analyzed using the log-rank test. Univariate Cox regression analysis was performed to identify variables that significantly impacted survival. Variables with a *P* value <0.05 in the univariate analysis were included in the multivariate analysis. The multivariate analysis estimated the adjusted hazard ratio (HR) of each variable, which represents the independent impact of each variable on survival. The prognostic abilities of the biomarkers were compared by time-dependent receiver operating characteristic (t-ROC) curves using the “time-ROC” R package [18]. The results of stratified survival analyzes were discussed based on clinicopathological characteristics. A two-tailed *P* < 0.05 was considered statistically significant. All statistical analyzes were determined using R software (version 3.6.3, https://www.r-project.org).

## 3. Results

### 3.1. Patient Characteristics

Clinicopathological characteristics of 57 colorectal cancer (CRC) patients: 49 male and 8 female, are shown in Table S2. The median patient age at the time of surgery was 60 [range from 25 to 80 years]. The median body mass index (BMI) was 20.9, ranging from 15.9 to 28.5. There were 7 patients (12.3%) with hypertension history, 6 (10.5%) with diabetes, 2 (3.5%) with tuberculosis infection, 11 (19.3%) with hyperlipidemia, and 9 patients (15.8%) with intestinal obstruction before surgery. There were 10 patients (17.5%) with a history of smoking, and 7 (12.3%) had a history of alcohol abuse. There were 4 (7.0%) patients who were received neoadjuvant treatment and 34 (59.6%) patients with postoperative adjuvant therapy. The median duration time of human immunodeficiency virus (HIV) infection was 5 months. The median treatment time was 4 months. The preoperative test displayed a mean CD4^+^ T lymphocyte count of 291.3 cell/*µ*L and a CD8^+^ T lymphocyte count of 667.3 cell/*µ*L. Based on the postoperative pathology, 12 patients (21.1%) had a tumor in the right colon, 26 patients (45.6%) had a tumor in the left colon, and 19 (33.3%) patients in rectum (including nine patients with anal squamous cell carcinoma). According to the eighth AJCC TNM staging system, 9 (15.8%), 25 (43.9%), 13 (22.8%), and 10 (17.5%) of patients had postoperative stages I, II, III, and IV, respectively.

### 3.2. Relationship between NLR and Clinicopathological Variables in Patients with HIV-Infected CRC

The relationship between clinicopathological variables and NLR is given in Table 1. There was no significant correlation between NLR and the following variables, but a higher NLR indicated a lower BMI (19.9 vs. 21.3, *P*=0.061) and increased tendency for lymph node metastasis (52.9% vs. 27.5%, *P*=0.078) and advanced stage (III-IV: 58.8% vs. 33.5%, *P*=0.082). Besides the neutrophil and lymphocyte counts, the mean value of CD8^+^ T lymphocytes was significantly higher in the low NLR group (758.8 cells per *µ*L) than in the high NLR group (457.6 cells per *µ*L). There was no significant difference in CD4^+^ T lymphocyte counts, resulting in higher CD3^+^ T lymphocyte counts in the low NLR group (1102.9 cells per *µ*L) than in the high NLR group (759.8 cells per *µ*L).

### 3.3. Prognosis Evaluation of Inflammation Biomarkers and Clinicopathological Features in HIV-Infected CRC Patients

**Figure 1:**
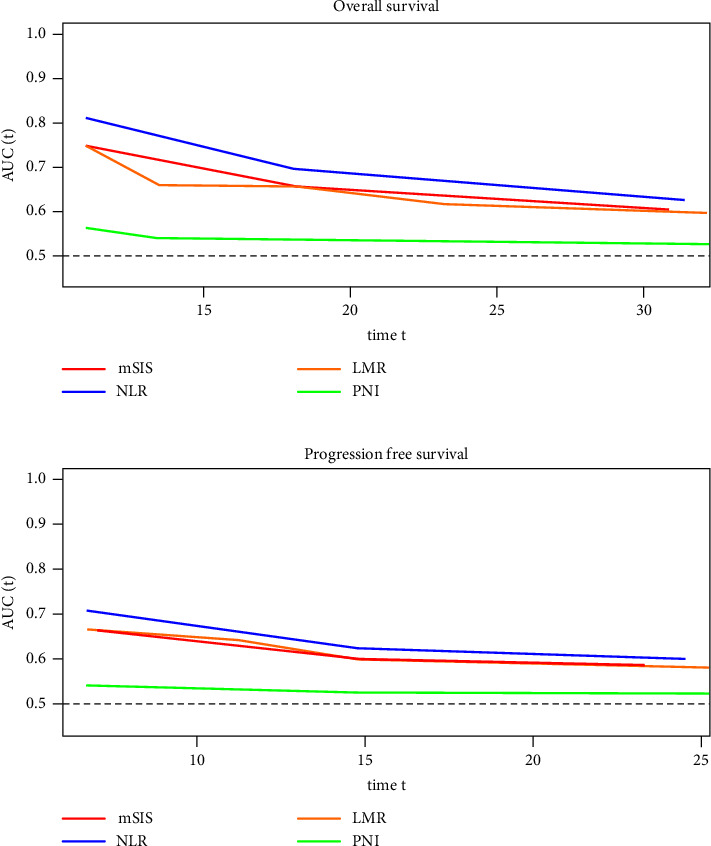
Time-dependent ROC curves for the systemic inflammation biomarkers. (a) AUC of each biomarker in over-all survival. (b) AUC of each biomarker in progression free survival. NLR: neutrophil-lymphocyte ratio; LMR: lymphocyte-monocyte ratio; PNI: prognostic nutritional index; mSIS: modified systemic inflammation score. The *x*-axis (time) means months after surgery.

The follow-up time of overall survival (OS) and progression-free survival (PFS) were 3 to 86 months. The cumulative three-year OS rate was 55.0%, and the cumulative three-year PFS rate was 45.0%. The univariate analysis presented the following clinicopathological indicators associated with OS: intestinal obstruction, TNM stage, CA125 level, CD8^+^ T lymphocyte count, NLR, LMR, and mSIS (Alb level <40 g/L and LMR < 3.0) (*P* < 0.05, Table 2). In multivariate analyzes, NLR ≤ 2.8 (HR: 0.094, 95% CI: 0.02–0.45), CD8^+^ T lymphocyte counts ≤912 per *µ*L (HR: 0.198, 95% CI: 0.044–0.9), III-IV stage (HR: 13.633, 95% CI: 3.824–48.603), and intestinal obstruction (HR: 5.872, 95% CI: 1.369–25.18) were independently related with OS (both *P* < 0.05, Table 2). The prognostic effect of NLR, LMR, PNI, SIS, mSIS, intestinal obstruction, tuberculosis infection, TNM stage, and CA125 level was also significantly related to PFS in univariate analysis (Table 3). In multivariate analysis of PFS, NLR ≤ 2.8 (HR = 0.265, 95% CI: 0.088–0.8), III-IV stage (HR: 8.242, 95% CI: 2.654–25.596), and intestinal obstruction (HR: 4.453, 95% CI: 1.116–17.771) were independently associated with PFS outcomes (both *P* < 0.05, Table 3). In the multivariate analysis, NLR was found to be the most significant biomarker compared to the other five biomarkers. All covariates satisfied the assumption of hazard proportionality (Figures S1 and S2). This study also used the time-ROC method to measure the prognostic value of each inflammation marker that was preoperatively available. In both OS and PFS, the time-ROC curve of NLR was consistently better than other indexes throughout the observation period (Figure 1). Comparing the OS after one, two, and three years of follow-up, the AUC values of NLR (0.81, 0.64, and 0.61) were higher than those of mSIS (0.75, 0.61, and 0.59), LMR (0.75, 0.61, and 0.59), and PNI (0.56, 0.53, and 0.52). Due to the small number of cases, we were unable to obtain statistical significance. However, NLR demonstrated a tendency to have better predictability in HIV-positive CRC patients.

### 3.4. Assessment of NLR Subgroups on OS and PFS Based on Clinicopathological Features

**Figure 2:**
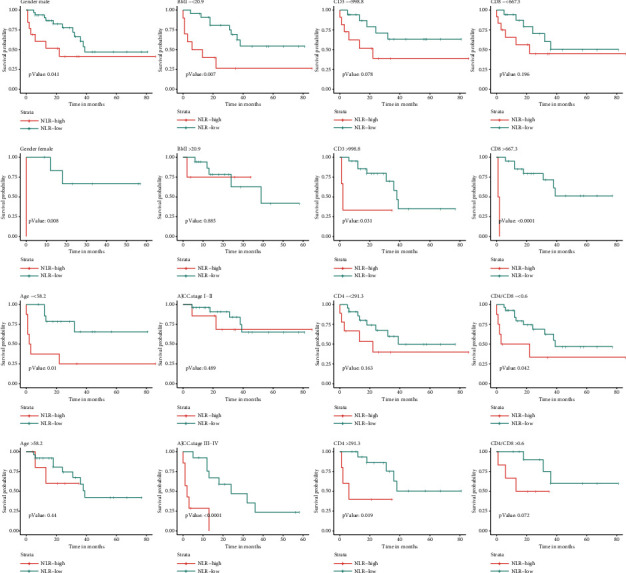
Analysis of OS based on gender, age, BMI, AJCC stage, CD3+ T lymphocytes counts, CD4+ T lymphocytes counts, CD8 T lymphocytes counts, and CD4/CD8 ratio in NLR subgroups.

**Figure 3:**
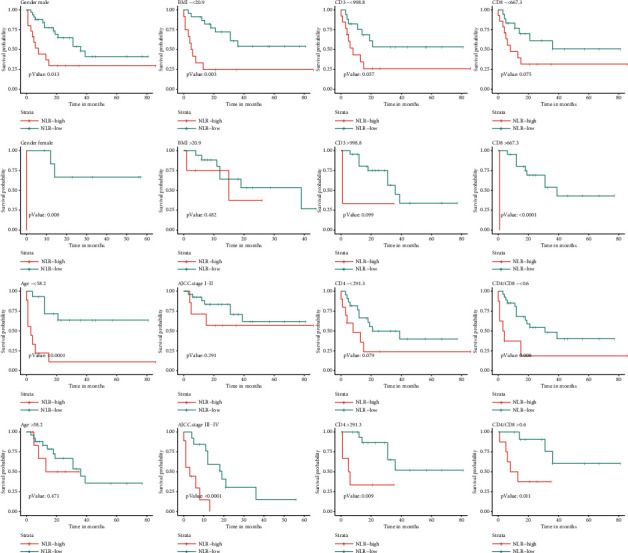
Analysis of PFS based on gender, age, BMI, AJCC stage, CD3+ T lymphocytes counts, CD4+ T lymphocytes counts, CD8+ T lymphocytes counts, and CD4/CD8 ratio in NLR subgroups.

According to the cut-off value of NLR, the OS and PFS subgroups of HIV-infected CRC patients were assessed. In CRC subgroup evaluation, the Kaplan–Meier curve exposed that gender (male and female), age (younger ≤58.2 years), BMI ≤ 20.9, stage III-IV, CD3^+^ T lymphocyte count >998.8 cells per *µ*L, CD8^+^ T lymphocyte count >667.3 cells per *µ*L, CD4^+^ T lymphocyte count >291.3 cells per *µ*L, and CD4/CD8 ≤ 0.6 in the high NLR group (>2.8) were closely associated with poor OS (Figure 2). In addition, gender (male and female), age (younger ≤ 58.2 years), BMI ≤ 20.9, stage III-IV, CD8^+^ T lymphocytes count >667.3 cells per *µ*L, CD4^+^ T lymphocytes count >291.3 cells per *µ*L, and CD4/CD8 (≤0.6 and >0.6) in the high NLR group (>2.8) were also associated with a poor PFS rate (Figure 3).

## 4. Discussion

This study investigated the prognostic value of systemic inflammation biomarkers in HIV-infected colorectal cancer (CRC) patients. We found that a high neutrophil-to-lymphocyte ratio (NLR) was significantly associated with shorter overall survival (OS) and progression-free survival (PFS) in surgically treated HIV-infected CRC patients compared to those with a low NLR. Multivariate analysis showed that NLR was an independent factor influencing survival outcomes. Our findings suggest that NLR can serve as a useful biomarker for surgical selection and prognosis of CRC patients infected with HIV.

In the past, individuals living with HIV (PLWH) had a higher incidence of certain malignancies such as non-Hodgkin lymphoma, Kaposi's sarcoma, anal cancer, and lung cancer, which were typically caused by opportunistic pathogens [19]. However, with the advancement of combination antiretroviral therapy (cART), non-HIV-related malignancies, including primary cancers in the liver, colorectal, breast, and prostate, have become a significant proportion of risk factors for life expectancy in HIV-infected people [20]. Studies conducted in Europe [21], North America [22], and Africa [23] have shown that HIV infection does not increase the incidence of colorectal cancer (CRC). However, there has been an increased expression of inhibitory immune checkpoint, such as CTLA-4, on the surface of T cells which suppresses the immunological attack against tumor cells [24]. Continuous HIV antigen stimulation persists even in patients receiving antiretroviral therapy, resulting in the tumor-specific CD8+ T cells becoming positive cells of CTLA-4 through the influence of continuously activated HIV-specific CD8+ T cells [25]. Although our previous study found no difference in immune checkpoint expression between HIV-infected CRC and normal patients [13], these controversial clinical findings suggest a need for more interest in the relationship between HIV-associated immunodeficiency and CRC development. However, some systematic reviews have discovered that PLWH with colorectal cancer are less likely to receive cancer screening or regular treatment, resulting in higher mortality rates than HIV-negative patients [26, 27]. The three-year overall survival (OS) rate in this cohort was only 55%, which is far below the average [1]. Additionally, our previous study found that colorectal cancer patients with HIV were more likely to be diagnosed at an advanced stage than CRC patients without HIV [13]. Therefore, simple and easily achievable prognostic predictors for patients with HIV-infected CRC are required. The earlier the subgroup of patients with a poorer prognosis can be identified, the sooner intervention and follow-up can be provided. The preoperative systemic inflammation biomarker is reliable in various cancers, making it worth investigating in HIV-positive CRC patients.

The neutrophil-to-lymphocyte ratio (NLR) is a common biomarker used to predict colorectal cancer prognosis [9]. Neutrophils secrete cytokines and chemokines that play a crucial role in cancer progression, while lymphocytes are involved in tumor antigen presentation and tumor cell killing. In non-HIV-infected CRC patients, low circulating lymphocytes are associated with poor prognosis [28, 29]. Several large-scale studies involving over 1000 CRC patients have found that a low preoperative NLR is an independent predictor of better survival outcomes [30–34]. The cut-off value of NLR reported in previous studies ranged from 2 to 3.75, with a mean value of 3, which is consistent with the current study. A preoperative NLR lower than 2.8 independently predicted better OS and PFS in a multivariate analysis of HIV-infected CRC patients. Several studies have shown that circulating monocytes in cancer patients' exhibit functional changes and that increased circulating monocytes are related to poorer OS in colorectal cancer patients. In previous CRC studies, lower lymphocyte counts and higher monocyte counts were linked to worse outcomes [30, 33, 35]. The current study found that an LMR below 3.0 was associated with poor OS and PFS but was not significant in multivariate analysis. The serum albumin indicates cancer patients' nutritional status and inflammation, impacting tumor progression. The prognostic nutritional index (PNI) combining serum albumin and lymphocyte at a low level (<45) negatively affected the OS and PFS in CRC patients [14, 36]. PNI < 45.7 was linked with worse PFS of HIV-infected CRC in univariate analysis but had no difference in OS. In recent years, a new prognostic biomarker, SIS, combining serum albumin and LMR, was explored in CRC patients. Suzuki et al. reported that increased SIS was independently related to poor prognosis [15]. However, the optimal LMR cut-off value in most studies was lower than the SIS definition value. Some studies modified SIS according to the optimal LMR cut-off value to obtain mSIS scoring [16]. Consequently, the scores in the current study were adjusted based on the best LMR. The increased mSIS could predict better than the SIS in HIV-infected CRC, but they were only correlated to OS and PFS in univariate analysis (Tables 2 and 3). Platelets promote cancer progression in diverse ways in the tumor microenvironment [37], but PLR was not significantly associated with OS or PFS in HIV-infected CRC patients. On the other side, comprehensive molecular stratification of CRC has shown that patients with the mesenchymal subtype have worse relapse-free and overall survival [7]. However, there is still a deficiency of genomic data on HIV-positive CRC patients. Therefore, obtaining this data in further clinical research is critical to provide subtype-based targeted interventions. In this study, six systemic inflammation biomarkers were investigated, including NLR, in HIV-infected CRC patients. The significance of the survival data was visualized using the popular time-dependent ROC analysis (t-ROC) method [38, 39]. The results of the multivariate COX and t-ROC analyzes suggest that NLR is the most effective systemic inflammation biomarker for predicting outcomes in CRC patients with HIV.

This study investigated the relationship between NLR and clinicopathological features. The results exposed that NLR was not interrelated with several clinicopathological features, including duration of HIV infection, tumor location, histology, and mismatch repair (MMR) status. Although there was no statistical difference, a high percentage of postoperative lymph node metastasis (52.9% vs. 27.5%) and stage III-IV (58.8% vs. 32.5%) were found in the high NLR subgroup (Table 1). In the stratified analysis, NLR had better prognostic performance in the subgroups of younger, low BMI, and stages III-IV (Figures 2 and 3). It may depict that NLR was negatively allied with disease severity in HIV-infected CRC patients. Alternatively, tumors with high infiltrate of CD8^+^ T and CD4^+^ T lymphocytes had a better prognosis [40]. However, in HIV-infected patients, serum CD4^+^ T lymphocytes counts mainly reflected a probability of various opportunistic infections, which is not a good predictor of cancer survival prognosis [5]. Hence, low NLR was associated with high CD8^+^ T lymphocytes counts but not with CD4^+^ T lymphocytes counts and CD4/CD8 ratio (Table 1). Furthermore, NLR had better prediction in the subgroups with higher CD4^+^, and CD8^+^ T lymphocytes counts, implying immune system recovery (Figures 2 and 3). We found that adjuvant therapy did not improve patient outcomes (Tables 2 and 3). While chemotherapy agents appear to downregulate the expression of immune inhibitors in CRC [24], it is possible that some patients in this cohort did not receive the full range of adjuvant therapy. Due to potential drug interactions between chemotherapeutic agents and cARTs drugs, chemotherapy should be used with caution in CRC patients with HIV infection. Further clinical studies are necessary to explore the efficacy of adjuvant therapy.

This study has some limitations that should be noted. First, due to the lack of preoperative C-reactive protein (CRP) and fibrinogen data, these markers were not analyzed in this study. Second, the small number of patients and short follow-up time may have affected the precision of the results. Therefore, well-designed clinical trials are needed to validate the role of inflammation biomarkers in HIV-infected CRC patients. It is also important to note that this study included nine cases of anal squamous cell carcinoma, which has a different pathogenesis than colorectal adenocarcinoma and is associated with immunosuppressive opportunistic infection. While previous studies have shown that low CD4^+^ T lymphocytes promote anal squamous carcinoma progression but not colorectal adenocarcinoma progression [41], the cases included in this study were unable to receive curative chemoradiotherapy for various reasons and were treated with surgery as a salvage option. Although these cases were rare, they were included in the study to explore the relationship between inflammation markers, surgical treatment, and prognosis. Therefore, further studies with more cases are needed to better understand the differences in the future.

## 5. Conclusion

With the availability of combination antiretroviral therapy (cART), people living with HIV can now have a similar life expectancy to that of the general population. Our findings demonstrate that the preoperative neutrophil-to-lymphocyte ratio (NLR) reflects the immune status and is an independent predictor of both overall survival (OS) and progression-free survival (PFS) in HIV-infected colorectal cancer patients. This simple prognostic biomarker has the potential to be used as a preoperative risk stratification tool, which could enable the development of individualized treatment plans for patients with HIV and colorectal cancer.

## Figures and Tables

**Table 1:** Relationship between NLR subgroups and clinicopathological features in CRC patients living with HIV.

Clinicopathological features	Low NLR group (*n* = 40)	High NLR group (*n* = 17)	*P* values
Gender			0.413
Male	33 (82.5%)	16 (94.1%)	
Female	7 (17.5%)	1 (5.9%)	
Age			0.369
Mean (SD)	59.5 (10.9)	55.4 (15.1)	
Median [MIN, MAX]	61 [29, 80]	58 [25, 78]	
BMI			0.061
Mean (SD)	21.3 (2.5)	19.9 (1.8)	
Median [MIN, MAX]	20.9 [15.9, 28.5]	20.2 [16.1, 23.9]	
Hypertension			0.146
No	36 (90.0%)	14 (82.4%)	
Yes	4 (10.0%)	3 (17.6%)	
DM			0.657
No	35 (87.5%)	16 (94.1%)	
Yes	5 (12.5%)	1 (5.9%)	
TB			0.511
No	39 (97.5%)	16 (94.1%)	
Yes	1 (2.5%)	1 (5.9%)	
Smoking			0.253
No	31 (77.5%)	16 (94.1%)	
Yes	9 (22.5%)	1 (5.9%)	
Alcohol abuse			1
No	35 (87.5%)	15 (88.2%)	
Yes	5 (12.5%)	2 (11.8%)	
Intestinal obstruction			0.109
No	36 (90.0%)	12 (70.6%)	
Yes	4 (10.0%)	5 (29.4%)	
Hyperlipidemia			0.191
No	30 (75.0%)	16 (94.1%)	
Yes	10 (25.0%)	1 (5.9%)	
Duration of HIV infection			0.657
Mean (SD)	34.9 (49.8)	34.2 (48)	
Median [MIN, MAX]	5.5 [0, 240]	4 [0, 144]	
Duration of HIV treatment			0.913
Mean (SD)	33 (48.3)	31.5 (42.7)	
Median [MIN, MAX]	4.5 [0, 240]	4 [0, 120]	
Tumor location			1
Right	9 (22.5%)	3 (17.6%)	
Left	31 (77.5%)	14 (82.4%)	
Histology			1
Squamous cell carcinoma	6 (15.0%)	3 (17.6%)	
Adenocarcinoma	34 (85.0%)	14 (82.4%)	
MMR status			1
dMMR	6 (15.0%)	3 (17.6%)	
pMMR	34 (85.0%)	14 (82.4%)	
SBR			0.631
1	3 (7.5%)	0 (0.0%)	
2	32 (80.0%)	14 (82.4%)	
3	5 (12.5%)	3 (17.6%)	
R0			0.109
Yes	36 (90.0%)	12 (70.6%)	
No	4 (10.0%)	5 (29.4%)	
T stage			0.235
I	2 (5.0%)	1 (5.9%)	
II	7 (17.5%)	0 (0.0%)	
III	18 (45.0%)	8 (47.1%)	
IV	13 (32.5%)	8 (47.1%)	
N stage			0.078
Nonmetastasis	29 (72.5%)	8 (47.1%)	
Metastasis	11 (27.5%)	9 (52.9%)	
M stage			0.464
Nonmetastasis	34 (85.0%)	13 (76.5%)	
Metastasis	6 (15.0%)	4 (23.5%)	
AJCC stage			0.082
I-II	27 (67.5%)	7 (41.2%)	
III-IV	13 (33.5%)	10 (58.8%)	
CA125 (0–35 U/ml)			0.248
Normal	35 (87.5%)	12 (70.6%)	
Increased	5 (12.5%)	5 (29.4%)	
CA153 (0–32.3 U/ml)			1
Normal	36 (90.0%)	16 (94.1%)	
Increased	4 (10.0%)	1 (5.9%)	
CA199 (0–37 U/ml)			0.795
Normal	30 (75.0%)	14 (82.4%)	
Increased	10 (25.0%)	3 (17.6%)	
AFP (0.89–8.78 ng/ml)			0.879
Normal	38 (95.0%)	17 (100.0%)	
Increased	2 (5.0%)	0 (0.0%)	
CEA (0–5 ng/ml)			0.124
Normal	29 (72.5%)	8 (47.1%)	
Increased	11 (27.5%)	9 (52.9%)	
HGB (g/L)			0.675
Mean (SD)	120.3 (19.9)	123.2 (29.2)	
Median [MIN, MAX]	117.5 [60, 152]	130 [68, 189]	
PLT count (^*∗*^1000 per *μ*l)			0.583
Mean (SD)	192.3 (74.9)	234.6 (139.2)	
Median [MIN, MAX]	189 [51, 400]	195 [93, 627]	
NEUT count (^*∗*^1000 per *μ*l)			0.001
Mean (SD)	2.6 (0.8)	4.6 (2.5)	
Median [MIN, MAX]	2.6 [1.1, 5.2]	4 [1.3, 9.9]	
LYMPH count (^*∗*^1000 per *μ*l)			<0.001
Mean (SD)	1.5 (0.5)	1 (0.4)	
Median [MIN, MAX]	1.5 [0.6, 3]	1.1 [0.4, 1.8]	
MONO count (^*∗*^1000 per *μ*l)			0.291
Mean (SD)	0.4 (0.1)	0.5 (0.2)	
Median [MIN, MAX]	0.4 [0.1, 0.7]	0.4 [0.2, 1]	
ALB (g/L)			0.222
Mean (SD)	39.1 (4.2)	40.3 (4.3)	
Median [MIN, MAX]	39 [32.2, 47.8]	40.6 [30, 46.3]	
CD3 count (per *μ*l)			0.001
Mean (SD)	1102.9 (390.9)	759.8 (279.6)	
Median [MIN, MAX]	1076 [376, 2363]	715 [344, 1310]	
CD8 count (per *μ*l)			0.001
Mean (SD)	758.8 (374.3)	457.6 (184.7)	
Median [MIN, MAX]	695 [158, 1918]	458 [142, 931]	
CD4 count (per *μ*l)			0.209
Mean (SD)	305.3 (155.7)	259.2 (186)	
Median [MIN, MAX]	282 [43, 729]	230 [13, 806]	
CD4/CD8			0.154
Mean (SD)	0.5 (0.4)	0.6 (0.4)	
Median [MIN, MAX]	0.4 [0, 1.6]	0.6 [0, 1.8]	

DM: diabetes mellitus; TB: tuberculosis infection status; MMR: mismatch repair; SBR: Scarff-Bloom-Richardson score; NLR: neutrophil-lymphocyte ratio; AFP: alpha fetoprotein; CEA: carcinoembryonic antigen; HGB: hemoglobin; PLT: platelet; NEUT: neutrophil; LYMPH: lymphocyte; MONO: monocyte; ALB: albumin.

**Table 2:** Univariate and multivariate analyses of clinicopathologic variables and inflammation biomarkers in relation to OS in patients living with HIV for CRC.

Clinicopathological features	Univariate analysis		Multivariate analysis	
	HR (95% CI)	*P* values	HR (95% CI)	*P* values
Gender				
Female	Reference			
Male	0.966 (0.284–3.28)	0.955		
Age	0.989 (0.951–1.028)	0.572		
BMI	0.976 (0.799–1.193)	0.814		
Hypertension				
Yes	3.344 (0.911–12.273)	0.069		
DM				
Yes	1.639 (0.368–7.306)	0.517		
TB				
Yes	3.952 (0.896–17.429)	0.07		
Smoking				
Yes	0.628 (0.185–2.135)	0.456		
Alcohol abuse				
Yes	0.561 (0.074–4.249)	0.576		
Intestinal obstruction				
Yes	5.678 (2.137–15.087)	<0.001	5.872 (1.369–25.18)	0.017
Hyperlipidemia				
Yes	1.125 (0.409–3.095)	0.819		
Duration of HIV infection (months)	0.997 (0.986–1.009)	0.616		
Duration of HIV treatment (months)	0.996 (0.983–1.009)	0.537		
Tumor location				
Right	Reference			
Left	1.026 (0.308–0.474)	0.966		
Rectal	1.585 (3.413–5.3)	0.455		
Histology				
Squamous cell carcinoma	Reference			
Adenocarcinoma	0.602 (0.201–1.809)	0.366		
MMR status				
dMMR	Reference			
pMMR	0.841 (0.247–2.859)	0.782		
AJCC stage				
I-II	Reference		Reference	
III-IV	5.767 (2.305–14.427)	<0.001	13.633 (3.824–48.603)	<0.001
Adjuvant treatment				
No	Reference			
Yes	1.225 (0.488–3.076)	0.666		
CA125 (0–35 U/ml)				
Increased	3.209 (1.122–9.178)	0.03	0.5 (0.079–3.183)	0.463
CA153 (0–32.3 U/ml)				
Increased	1.808 (0.53–6.165)	0.344		
CA199 (0–37 U/ml)				
Increased	1.772 (0.708–4.434)	0.221		
CEA (0–5 ng/ml)				
Increased	1.23 (0.495–3.056)	0.656		
CD8 count (per *μ*l)				
>912	Reference		Reference	
≤912	0.342 (0.134–0.875)	0.025	0.198 (0.044–0.9)	0.036
CD4 count (per *μ*l)				
>106	Reference			
≤106	2.444 (0.822–7.271)	0.108		
CD4/CD8				
>0.6	Reference			
≤0.6	2.978 (0.693–12.801)	0.143		
NLR				
>2.8	Reference		Reference	
≤2.8	0.337 (0.138–0.823)	0.017	0.094 (0.02–0.45)	0.003
LMR				
>3.0	Reference		Reference	
≤3.0	5.024 (1.977–12.772)	<0.001	0.888 (0.125–6.329)	0.905
PLR				
>131.4	Reference			
≤131.4	2.079 (0.874–4.948)	0.098		
PNI				
>45.7				
≤45.7	1.953 (0.822–4.641)	0.13		
SIS				
0	Reference			
1	1.89 (0.401–8.919)	0.421		
2	2.941 (0.65–13.298)	0.161		
mSIS				
0	Reference		Reference	
1	3.089 (0.967–9.863)	0.057	2.016 (0.457–8.891)	0.354
2	7.232 (2.056–25.436)	0.002	5.926 (0.561–62.638)	0.139

DM: diabetes mellitus; TB: tuberculosis infection status; MMR: mismatch repair; NLR: neutrophil-lymphocyte ratio; LMR: lymphocyte-monocyte ratio; PLR: platelet-lymphocyte ratio; PNI: prognostic nutritional index; SIS: systemic inflammation score; mSIS: modified systemic inflammation score; CD8: CD8+ T lymphocytes; CD4: CD4+ T lymphocytes.

**Table 3:** Univariate and multivariate analysis of clinicopathologic variables and inflammation biomarkers in relation to PFS in patients living with HIV for CRC.

Clinicopathological features	Univariate analysis		Multivariate analysis	
	HR (95% CI)	*P* values	HR (95% CI)	*P* values
Gender				
Female	Reference			
Male	0.687 (0.207–2.284)	0.54		
Age	0.983 (0.953–1.014)	0.285		
BMI	0.99 (0.83–1.18)	0.906		
Hypertension				
Yes	2.339 (0.674–8.114)	0.181		
DM				
Yes	2.018 (0.596–6.825)	0.259		
TB				
Yes	4.756 (1.06–21.34)	0.042	2.035 (0.39–10.608)	0.399
Smoking				
Yes	0.781 (0.27–2.259)	0.648		
Alcohol abuse				
Yes	0.399 (0.054–2.96)	0.369		
Intestinal obstruction				
Yes	3.083 (1.278–7.437)	0.012	4.453 (1.116–17.771)	0.034
Hyperlipidemia				
Yes	0.71 (0.268–1.882)	0.492		
Duration of HIV infection (months)	0.999 (0.99–1.008)	0.799		
Duration of HIV treatment (months)	0.999 (0.989–1.008)	0.782		
Tumor location				
Right	Reference			
Left	1.023 (0.349–0.604)	0.968		
Rectal	1.742 (2.993–5.028)	0.305		
Histology				
Squamous cell carcinoma	Reference			
Adenocarcinoma	0.479 (0.192–1.197)	0.115		
MMR status				
dMMR	Reference			
pMMR	0.791 (0.273–2.289)	0.665		
AJCC stage				
I-II	Reference		Reference	
III-IV	4.901 (2.196–10.937)	<0.001	8.242 (2.654–25.596)	<0.001
Adjuvant treatment				
No	Reference			
Yes	1.583 (0.686–3.65)	0.281		
CA125 (0–35 U/ml)				
Increased	2.412 (1–5.815)	0.05	0.65 (0.173–2.449)	0.524
CA153 (0–32.3 U/ml)				
Increased	1.982 (0.681–5.765)	0.209		
CA199 (0–37 U/ml)				
Increased	1.365 (0.595–3.129)	0.462		
CEA (0–5 ng/ml)				
Increased	1.316 (0.599–2.894)	0.494		
CD8 count (per *μ*l)				
>912	Reference			
≤912	0.47 (0.201–1.098)	0.081		
CD4 count (per *μ*l)				
>106	Reference			
≤106	1.774 (0.613–5.136)	0.291		
CD4/CD8				
>0.6	Reference			
≤0.6	2.067 (0.714–5.982)	0.181		
NLR				
>2.8	Reference		Reference	
≤2.8	0.316 (0.145–0.688)	0.004	0.265 (0.088–0.8)	0.019
LMR				
>3.0	Reference		Reference	
≤3.0	5.546 (2.476–12.423)	<0.001	0.649 (0.065–6.47)	0.713
PLR				
>131.4	Reference			
≤131.4	1.38 (0.648–2.941)	0.404		
PNI				
>45.7			Reference	
≤45.7	2.33 (1.077–5.04)	0.032	0.957 (0.11–8.31)	0.968
SIS				
0	Reference		Reference	
1	2.211 (0.477–10.253)	0.311	2.523 (0.394–16.165)	0.329
2	4.785 (1.095–20.912)	0.037	1.438 (0.093–22.121)	0.795
mSIS				
0	Reference		Reference	
1	2.381 (0.879–6.446)	0.088	1.823 (0.39–8.52)	0.446
2	7.216 (2.551–20.412)	<0.001	7.179 (0.233–220.83)	0.259

DM: diabetes mellitus; TB: tuberculosis infection status; MMR: mismatch repair; NLR: neutrophil-lymphocyte ratio; LMR: lymphocyte-monocyte ratio; PLR: platelet-lymphocyte ratio; PNI: prognostic nutritional index; SIS: systemic inflammation score; mSIS: modified systemic inflammation score; CD8: CD8+ T lymphocytes; CD4: CD4+ T lymphocytes.

## Data Availability

The data used to support the findings of this study are available from the corresponding author upon request.

## Supplementary Materials

Supplementary Materials Table S1: The definition of SIS and modified SIS (mSIS). Table S2: Baseline clinicopathologic features and preoperative laboratory tests of CRC patients living with HIV. Figure S1: The proportional hazard test for each covariate included in the multivariate Cox analysis of OS. Figure S2: The proportional hazard test for each covariate included in the multivariate Cox analysis of PFS.
