# Supplementary material for: Higher Neutrophil-to-Lymphocyte Ratio (NLR) Is a Preoperative Inflammation Biomarker of Poor Prognosis in HIV-Infected Patients with Colorectal Cancer: A Retrospective Study
Source: Can J Gastroenterol Hepatol. 2023 Mar 6;2023:7966625. doi: 10.1155/2023/7966625 (PMC10010889; doi:10.1155/2023/7966625)
Supplement: Supplementary Materials — Table S1: The definition of SIS and modified SIS (mSIS). Table S2: Baseline clinicopathologic features and preoperative laboratory tests of CRC patients living with HIV. Figure S1: The proportional hazard test for each covariate included in the multivariate Cox analysis of OS. Figure S2: The proportional hazard test for each covariate included in the multivariate Cox analysis of PFS. [file 7966625.f1.docx]

**Table S1**

| The definition of SIS and modified SIS (mSIS) | |
| --- | --- |
|  |  |
| Scoring System | Score |
| The SIS |  |
| ALB (≥40 g/l ) and LMR (≥4.44 ) | 0 |
| ALB (≥40 g/l ) and LMR (<4.44 ) | 1 |
| ALB (<40 g/l ) and LMR (≥4.44 ) | 1 |
| ALB (<40 g/l ) and LMR (<4.44 ) | 2 |
| The mSIS |  |
| ALB (≥39.1 g/l ) and LMR (≥3.0) | 0 |
| ALB (≥39.1 g/l ) and LMR (<3.0) | 1 |
| ALB (<39.1 g/l ) and LMR (≥3.0 ) | 1 |
| ALB (<39.1 g/l ) and LMR (<3.0 ) | 2 |
|  |  |
| SIS: systemic inflammation score; LMR: lymphocyte-monocyte ratio; ALB: albumin | |

**Table S2**

| Baseline clinicopathologic features and preoperative laboratory tests of CRC patients living with HIV | | | |
| --- | --- | --- | --- |
|  |  |  |  |
|  | ALL (n=57) |  | ALL (n=57) |
| **Gender** |  | **CA125 (0-35U/ml)** |  |
| male | 49 (86.0%) | normal | 47 (82.5%) |
| female | 8 (14.0%) | increased | 10 (17.5%) |
| **Age** |  | **CA153 (0-32.3U/ml)** |  |
| Mean (SD) | 58.2 (12.3) | normal | 52 (91.2%) |
| Median [MIN, MAX] | 60 [25,80] | increased | 5 (8.8%) |
| **BMI** |  | **CA199 (0-37U/ml)** |  |
| Mean (SD) | 20.9 (2.4) | normal | 44 (77.2%) |
| Median [MIN, MAX] | 20.9 [15.9,28.5] | increased | 13 (22.8%) |
| **Hypertension** |  | **AFP (0.89-8.78ng/ml)** |  |
| no | 50 (87.7%) | normal | 55 (96.5%) |
| yes | 7 (12.3%) | increased | 2 (3.5%) |
| **DM** |  | **CEA (0-5ng/ml)** |  |
| no | 51 (89.5%) | normal | 37 (64.9%) |
| yes | 6 (10.5%) | increased | 20 (35.1%) |
| **TB** |  | **HGB (g/L)** |  |
| no | 55 (96.5%) | Mean (SD) | 121.2 (22.8) |
| yes | 2 (3.5%) | Median [MIN, MAX] | 120 [60,189] |
| **Smoking** |  | **PLT count (*1000 per uL)** |  |
| no | 47 (82.5%) | Mean (SD) | 204.9 (99.1) |
| yes | 10 (17.5%) | Median [MIN, MAX] | 195 [51,627] |
| **Alcohol abuse** |  | **NEUT count (*1000 per uL)** |  |
| no | 50 (87.7%) | Mean (SD) | 3.2 (1.7) |
| yes | 7 (12.3%) | Median [MIN, MAX] | 2.8 [1.1,9.9] |
| **Intestinal Obstruction** |  | **LYMPH count (*1000 per uL)** |  |
| no | 48 (84.2%) | Mean (SD) | 1.4 (0.5) |
| yes | 9 (15.8%) | Median [MIN, MAX] | 1.3 [0.4,3] |
| **Hyperlipidemia** |  | **MONO count (*1000 per uL)** |  |
| no | 46 (80.7%) | Mean (SD) | 0.4 (0.2) |
| yes | 11 (19.3%) | Median [MIN, MAX] | 0.4 [0.1,1] |
| **Duration of HIV infection (months)** |  | **ALB (g/L)** |  |
| Mean (SD) | 34.7 (48.9) | Mean (SD) | 39.4 (4.2) |
| Median [MIN, MAX] | 5 [0,240] | Median [MIN, MAX] | 39.2 [30,47.8] |
| **Duration of HIV treatment (months)** |  | **CD3 count (per ul)** |  |
| Mean (SD) | 32.6 (46.3) | Mean (SD) | 998.8 (392) |
| Median [MIN, MAX] | 4 [0,240] | Median [MIN, MAX] | 937 [344,2363] |
| **Tumor Location** |  | **CD8 count (per ul)** |  |
| right | 12 (21.1%) | Mean (SD) | 667.3 (355.3) |
| left | 26 (45.6%) | Median [MIN, MAX] | 559.5 [142,1918] |
| rectal | 19 (33.3%) | **CD4 count (per ul)** |  |
| **Histology** |  | Mean (SD) | 291.3 (165.1) |
| squamous cell carcinoma | 9 (15.8%) | Median [MIN, MAX] | 274 [13,806] |
| adenocarcinoma | 48 (84.2%) | **CD4/CD8** |  |
| **MMR status** |  | Mean (SD) | 0.6 (0.4) |
| dMMR | 9 (15.8%) | Median [MIN, MAX] | 0.5 [0,1.8] |
| pMMR | 48 (84.2%) | **NLR** |  |
| **SBR** |  | Mean (SD) | 2.7 (1.8) |
| 1 | 3 (5.3%) | Median [MIN, MAX] | 2.1 [0.9,9.5] |
| 2 | 46 (80.7%) | **LMR** |  |
| 3 | 8 (14.0%) | Mean (SD) | 4 (2.4) |
| **R0** |  | Median [MIN, MAX] | 3.7 [1.2,16.7] |
| 0 | 48 (84.2%) | **PLR** |  |
| 1 | 9 (15.8%) | Mean (SD) | 171.2 (121.4) |
| **T stage** |  | Median [MIN, MAX] | 142.6 [44,829.1] |
| I | 3 (5.3%) | **PNI** |  |
| II | 7 (12.3%) | Mean (SD) | 46.3 (4.6) |
| III | 26 (45.6%) | Median [MIN, MAX] | 46.6 [33.3,55.6] |
| IV | 21 (36.8%) | **SIS** |  |
| **N stage** |  | 0 | 9 (15.8%) |
| 0 | 37 (64.9%) | 1 | 24 (42.1%) |
| I | 10 (17.5%) | 2 | 24 (42.1%) |
| II | 10 (17.5%) | **mSIS** |  |
| **M stage** |  | 0 | 23 (40.4%) |
| 0 | 47 (82.5%) | 1 | 24 (42.1%) |
| I | 10 (17.5%) | 2 | 10 (17.5%) |
| **AJCC stage** |  | **Neoadjuvant therapy** |  |
| I | 9 (15.8%) | yes | 4 (7.0%) |
| II | 25 (43.9%) | **Adjuvant therapy** |  |
| III | 13 (22.8%) | yes | 34 (59.6%) |
| IV | 10 (17.5%) |  |  |
|  |  |  |  |
| DM: diabetes mellitus; TB: tuberculosis infection status; MMR: mismatch repair; SBR: Scarff-Bloom-Richardson score; R0: radical resection; AFP: alpha fetoprotein; CEA: carcinoembryonic antigen; HGB: hemoglobin; PLT: platelet; NEUT: neutrophil; LYMPH: lymphocyte; MONO: monocyte; ALB: albumin; NLR: neutrophil-lymphocyte ratio; LMR: lymphocyte-monocyte ratio; PLR: platelet-lymphocyte ratio; PNI: prognostic nutritional index; SIS: systemic inflammation score; mSIS: modified systemic inflammation score | | | |

**
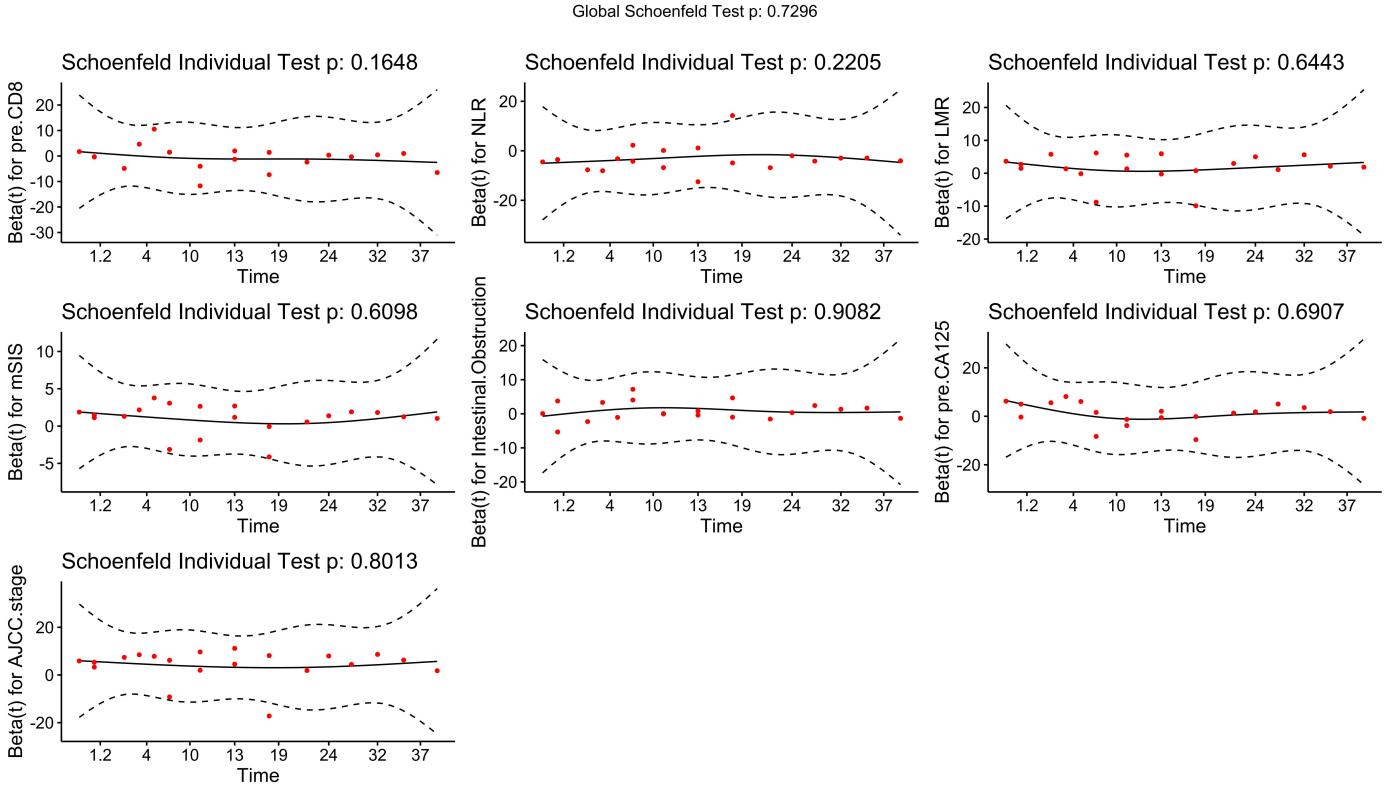
**

**Figure S1:** The proportional hazard test for each covariate included in the multivariate Cox analysis of OS.


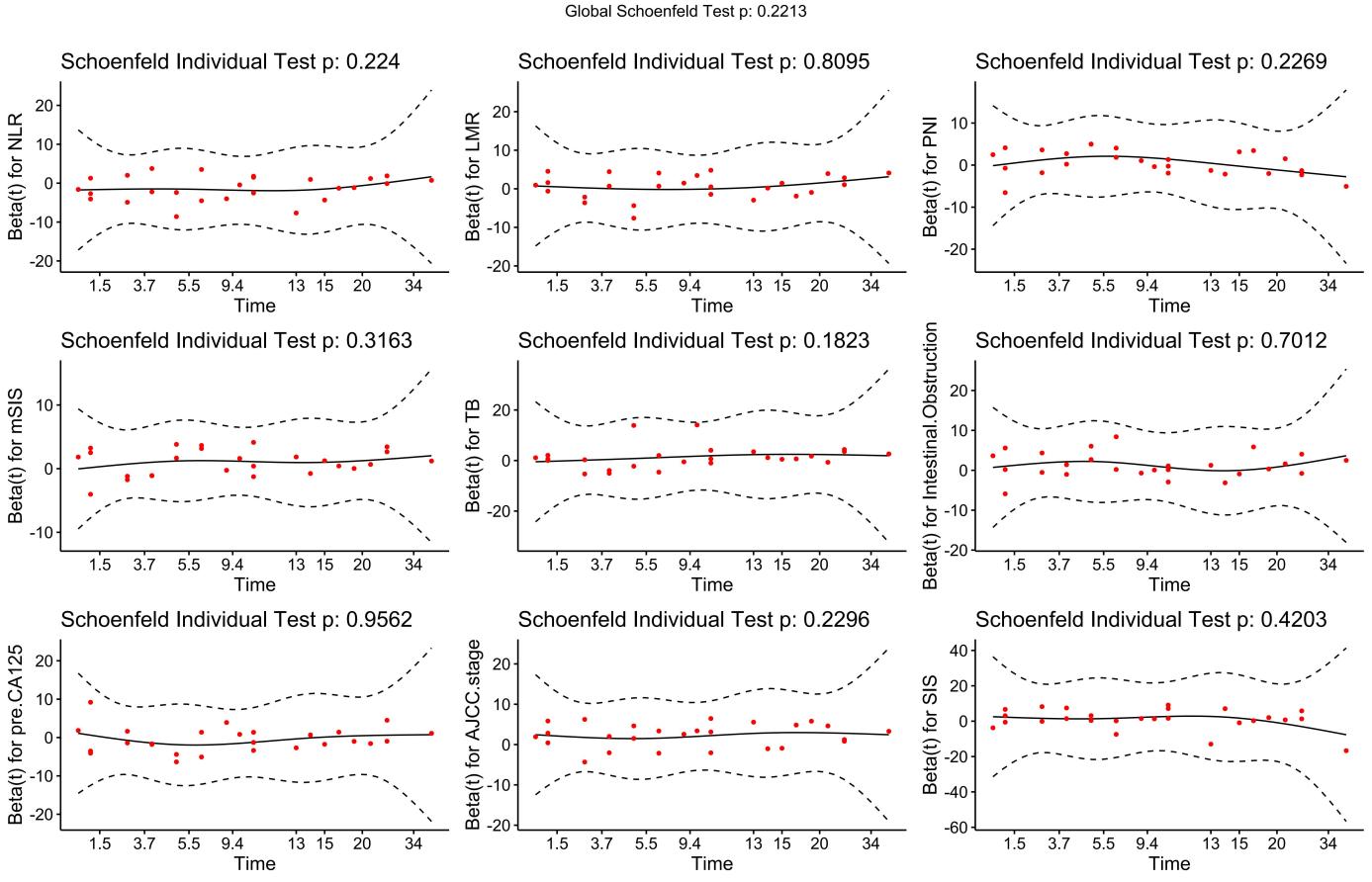


**Figure S2:** The proportional hazard test for each covariate included in the multivariate Cox analysis of PFS.
